# Does Autoimmunity Play a Role in the Immunopathogenesis of Vasculitis Associated With Chronic Chagas Disease?

**DOI:** 10.3389/fcimb.2021.671962

**Published:** 2021-07-06

**Authors:** Victor Garcia-Bustos, Pedro Moral Moral, Marta Dafne Cabañero-Navalon, Miguel Salavert Lletí, Eva Calabuig Muñoz

**Affiliations:** ^1^ Department of Internal Medicine and Infectious Diseases, University and Polytechnic La Fe Hospital, Valencia, Spain; ^2^ Department of Medicine, University of Valencia, Valencia, Spain

**Keywords:** Chagas, *Trypanosoma cruzi*, vasculitis, autoimmunity, immunopathology

## Introduction

Chagas disease (CD) is a chronic systemic vector-borne infection caused by the protozoan *Trypanosoma cruzi*. It has spread from Latin America through migration, becoming a global issue (Pérez-Molina and Molina, 2018). Its prevalence is ∼7 million people worldwide, of whom 30-40% will develop severe chronic complications such as cardiomyopathy or megaviscerae, with a considerable impact on morbimortality (WHO, 2020; WHO, 2021).

The parasite is transmitted after metacyclic trypomastigotes in the feces of a triatomine insect enter the host through the bite wound. They penetrate cells and transform into amastigotes, where they multiply by binary fission and differentiate again into circulating trypomastigotes after rupture of the host cell. The triatomine vector ingests them from an infected individual and they replicate in its intestine in the form of epimastigotes. The cycle closes after the epimastigotes differentiate again into metacyclic trypomastigotes (Pérez-Molina and Molina, 2018).

Chronic clinical manifestations in CD have been associated with a disproportionate inflammation compared to the parasitic burden (Cunha-Neto et al., 2011), driven by direct parasitic invasiveness (Bonney and Engman, 2008; Epting et al., 2010), damage to bystander cells (De Bona et al., 2018), cellular injury due to non-specific immune responses (Bellotti et al., 1996; Zhang and Tarleton, 1999; Bonney and Engman, 2015; De Bona et al., 2018), autoimmunity (Leon et al., 2001; Engman and Leon, 2002; Bonney and Engman, 2015; De Bona et al., 2018), and vasculitis (Roffê et al., 2016; Weaver et al., 2019).

Herein, we review the autoimmune mechanisms behind chronic CD and, particularly, the knowledge gaps in the immunopathogenesis of vasculitis developed in patients with CD.

## What We Know About Autoimmunity in Chronic Chagas Disease

Since the first insights into the pathophysiology of CD, autoimmunity has been attributed a potential key role in the development of chronic complications of the disease. The scarcity of viable parasites in chronic Chagas cardiomyopathy (CCC) contrasts with the severity of the disease (Cunha-Neto et al., 2011). Some studies have not found a correlation between the parasitic burden and the degree of tissue inflammation (Dias et al., 1956; Todorov et al., 2003). The identification of a relatively large number of autoantibodies, autoreactive T cells, and myocardial specific antigens as targets of autoreactive responses (Rizzo et al., 1989; Leon et al., 2001; Engman and Leon, 2002) suggests that the immune response is not completely pathogen-specific. However, despite the presence of diverse autoimmunity processes has been extensively described in several studies, their pathogenic role has yet to be clarified.

The current autoimmune hypothesis suggests that the initial parasite-triggered cardiac damage leads to a release of self-antigens within an inflammatory environment (Engman and Leon, 2002), which leads to a breakdown of self-tolerance as a consequence of the potent immune stimuli. Owing to molecular mimicry between parasite and host protein epitopes within the microinflammatory environment caused by parasitic invasion (Cunha-Neto et al., 1996), polyclonal B cell and bystander activation (De Bona et al., 2018) and cross-activation of autoreactive T or B cells to host proteins is produced (Iwai et al., 2005). This triggers the synthesis of autoantibodies targeted to multiple antigens (De Bona et al., 2018). The parasite persistence in chronic stages of the infection causes a certain degree of myocytolysis and oxidative stress (Wen et al., 2004). Furthermore, toxin and bioactive lipids release, microvascular changes, and T cell-mediated delayed-type hypersensitivity, perpetuate the damage and the immune vicious circle (Leon et al., 2001; Engman and Leon, 2002; Bonney and Engman, 2015). Recently, important local structural and functional alterations have been described as a consequence of thymic infection by *T. cruzi*, which produces abnormal thymocyte migration and a disrupted negative selection of the T-cell repertoire (Pérez et al., 2020). Secondarily, abnormal activated double-positive and double-negative lymphocytes with a proinflammatory phenotype have been found in patients with CD. This has been proposed to contribute to dysimmunity as a pathogenic determinant of chronic *T. cruzi* cardiac infection (Morrot et al., 2011; Flávia Nardy et al., 2015; Passos et al., 2017).

Several *T. cruzi* antigens have been demonstrated to cross-react with human molecules and generate autoantibodies that may be implicated in CCC, as seen in **Table 1**. Furthermore, in patients with CCC, the presence of anti-β1-adrenergic receptors and anti-M2 receptors has been associated with progression (Retondaro et al., 1999; Labovsky et al., 2007); and, in the last, to electrical instability and sudden death (Medei et al., 2007). The existence a robust autoreactive T cell reaction feeds back the production of autoantibodies and suggests that autoimmune and dysimmune processes play a significant role in the pathogenesis.

**Table 1:** Cross-reactivity between *T. cruzi* and human antigens with possible implications in Chagasic cardiomyopathy.

*Trypanosoma cruzi* antigen	Human antigen	Reference
Glycosphingolipids	Neutral glycosphingolipids	Vermelho et al., 1997
Cruzipain	Cardiac muscarinic acetylcholine receptors (M2)	Goin et al., 1999; Acosta et al., 2011
23KDa ribosomal protein	Ribosomal P protein	Bonfa et al., 1993; Kaplan et al., 1997
Sarcoplasmic reticulum adenosine-triphosphatase (SRA)	SRA of cardiomyocytes and skeletal muscle myocytes	Santos-Buch et al., 1985
Microsomal fraction (Mc)	Cha autoantigen	Laucella et al., 1996
Shed acute-phase antigen (SAPA)	Galectin-1	Giordanengo et al., 2001
Ribosomal P0 and P2β proteins	Cardiac β1 adrenergic receptor	Labovsky et al., 2007; Abraham and Derk, 2015; Jiménez et al., 2017
B13 protein	Cardiac myosin heavy chain	Cunha-Neto et al., 1996

Inflammatory responses mediated both by T CD4+ and CD8+ cells (Tarleton, 2001; Cunha-Neto et al., 2011), antibody-dependent cell-mediated cytotoxicity (ADCC) (Bonney and Engman, 2008) and complement activation with formation of the membrane attack complex (MAC) (Aiello et al., 2002) are proposed factors for autoimmune-induced cell damage. After bystander activation and release of proinflammatory cytokines and mediators (such as tumor necrosis factor (TNF), interferon-gamma (IFN-γ), bradykinin, prostaglandins, endothelin, or nitric oxide, among other) and reactive oxygen species (ROS) by cells of the innate immune system, these specific autoantibodies interact with neutrophils, eosinophils, and NK cells *via* CD16, and perforins, proteolytic enzymes or TNF are released (De Bona et al., 2018). Furthermore, the complement system becomes activates and the MAC is assembled on cardiomyocytes and endothelial cells, which result in muscle injury and vasculitis (Aiello et al., 2002). Interestingly, significantly increased levels of circulating activated T cells and CD5+ B cells have been found in patients with CCC. This pattern has also been described in patients with rheumatoid arthritis and multiple sclerosis, where autoimmune, polyclonal and hyperimmune responses are responsible for the pathogeny of the disease (Dutra et al., 1994).

However, whether autoimmunity is determinant for the pathophysiology of chronic complications of the disease or is just an epiphenomenon is not clear. Some studies advocate the idea that autoimmune responses may be present in absence of inflammation, or be a consequence and not a cause of tissue injury (Leon and Engman, 2003). Others state pathogen-specific type 1 immune responses and not autoimmunity could be related to tissue injury (Roffê et al., 2016), and many keep the debate and controversy about the autoimmune hypothesis due to experimental limitations, low result reproducibility, and lack of persuasive evidence that autoreactive responses truly effect the observed injury in the multifaceted pathogeny of CCC (Levin, 1996; Kierszenbaum, 2003; Girones et al., 2007; Bonney and Engman, 2008; Tanowitz et al., 2009; Cunha-Neto et al., 2011). Interestingly, some of the antigens which have shown cross-reactivity with human epitopes, such as cruzipain as the most important one, are candidates for vaccine against *T. cruzi* (Bivona et al., 2020). Despite vaccine promotion was limited for years because of the possibility to induce autoimmunity, posterior evidence suggested autoimmunity as a consequence of parasite persistence and prioritized the parasite clearance to avoid it (Bonney and Engman, 2015; Bivona et al., 2020). In fact, immunization with different cruzipain analogues has demonstrated protective immunity without causing cardiac abnormalities (Cazorla et al., 2015).

## Vasculitis in the Pathophysiology of Chronic Chagas Disease: The Great Forgotten?

Different pathogenic mechanisms have been described to explain the large variability in the clinical outcome of *T. cruzi* infection. In addition to direct parasite damage (Bonney and Engman, 2008; Epting et al., 2010), bystander effect (De Bona et al., 2018), autoimmunity (Leon et al., 2001; Engman and Leon, 2002; Bonney and Engman, 2015; De Bona et al., 2018), and non-specific immune responses (Bellotti et al., 1996; Zhang and Tarleton, 1999; Bonney and Engman, 2015; De Bona et al., 2018), the presence of vasculitis in chronic CD has long been reported. However, contrarily to the previous factors, the immune mechanisms driving vasculitis and its pathogenic implications have hardly been studied.

*T. cruzi* infection has been found to cause vasculitis, perivascular inflammation, vascular necrosis, and endothelitis affecting both macrovasculature and microvasculature of several organs and tissues (Dias et al., 1956; Okumura et al., 1960; Cossermelli et al., 1978; Sunnemark et al., 1998; Petkova et al., 2001; Tanowitz et al., 2009; Prado et al., 2011; Roffê et al., 2016; Weaver et al., 2019). Coronary vessels (Petkova et al., 2000; Petkova et al., 2001), aorta (Petkova et al., 2000; Petkova et al., 2001, liver (Mukherjee et al., 2003), skeletal muscle (Roffê et al., 2016; Weaver et al., 2019), and nerves blood vessels (Said et al., 1985; González Cappa et al., 1987) are involved as demonstrated by studies in several animal models, including mice, pigs, and dogs. Recent works in murine models have demonstrated that its implication in the pathogeny of *T. cruzi* infection might be determinant, with low-level parasite persistence driving severe paralyzing systemic necrotizing vasculitis (Roffê et al., 2016; Weaver et al., 2019).

In humans, the first description of vasculitis was in 1911 (Vianna, 1911). Cerebral vasculitis (Petkova et al., 2001), muscle vasculitis (Laguens et al., 1975; Cossermelli et al., 1978), and obliterative coronary vasculitis (Dias et al., 1956) have been reported. Moreover, recently, our group published the first case of a patient with anti-neutrophil cytoplasmic antibody-positive (ANCA) vasculitis and chronic CD (Garcia-Bustos et al., 2020a). This case was also a highly atypical presentation of ANCA-associated vasculitis, as presented with periaortitis, possibly due to vasa vasorum vasculitis, and muscle vasculitis, as described in the murine model. This again raises the dilemma of the pathophysiology of vasculitis in chronic CD and the potential role and implication of autoimmunity in its development.

Some studies reported endothelial invasion by amastigotes of *T. cruzi* in the very early stages of infection, even before parasitemia occurs (Factor et al., 1985). However, this does not explain the inflammatory involvement of many vascular beds in the late stages of the disease when the parasite burden is practically non-existent. Nevertheless, parasitic endothelial invasion has been demonstrated to induce expression of proinflammatory cytokines, nitric oxidase synthases, and adhesion molecules (Tanowitz et al., 1992; Sunnemark et al., 1998; Campos-Estrada et al., 2015). Some vasoactive mediators such as bradykinin, endothelin-1, and thromboxane A2 (Prado et al., 2011; Garcia-Bustos et al., 2020b) have also been implicated in the inflammatory process. These findings would make us hypothesize whether the interaction between the endothelium and effector immune cells within an inflammatory environment may induce bystander activation mechanisms, molecular mimicry, and development of autoreactive T cells and antibodies.

Despite the many advances made in the cellular and molecular immune mechanisms of chronic CD during the 80s and 90s, only recently have we been able to know the first glimpses of the immunological mechanisms underlying the development of Chagas vasculitis. In a murine model, Roffê, Weaver, and coworkers (2016 and 2019, respectively) described systemic necrotizing vasculitis with histological findings resembling human polyarteritis nodosa (PAN), with skeletal muscle arteries being the most severely affected. The presence of large inflammatory infiltrates composed by F4/80+ myeloid cells and *T. cruzi* tetramer-specific CD8+ T lymphocytes producing tumor necrosis factor-alpha (TNF-α), interferon-gamma (IFN-γ) but not interleukin 17 (IL-17) without neutrophils or immune complexes, advocates for a pathogen-specific type 1 immune response and not autoimmunity as the pathogenic mechanism. In concordance with other findings (González Cappa et al., 1987), no parasites were observed, so the severity of vasculitis is highly disproportionate to the low parasite burden. Hence, whether the recruitment of autoreactive responses after a parasite-induced breakdown of self-tolerance triggers or contributes to the Chagas vasculitis immunopathology is yet to be determined.

## Autoimmunity and Chagas Vasculitis. What if…?

There is a large variability in the outcomes of CD due to its possibility to infect several hosts and diverse behavior within, parasite diversity, and different immunopathogenic mechanisms of disease (De Bona et al., 2018). In the same way, different vasculitis patterns have been described both in animal models and humans associated with CD.

Primary systemic vasculitides have been traditionally classified according to the main vessels’ caliber involved: large-vessel, such as giant cell arteritis or Takayasu arteritis; medium-vessel, such as PAN or Kawasaki disease, and small-vessel vasculitides. The latter can be subdivided into ANCA-associated vasculitis (AAV) and immune complex small vessel vasculitis (Shavit et al., 2018). Their pathogenesis is complex and diverse, and genetic and immunological determinants have been identified (Ozen and Batu, 2018). However, a key feature of AAV which is not present in other vasculitides is the presence of ANCA directed against myeloperoxidase (MPO) or proteinase-3 (PR3), which are determinants for their pathogenesis (Leacy et al., 2020). In CD, many vascular beds are affected, with histology mainly showing mononuclear and macrophage infiltration, fibrinoid necrosis, and necrotizing vasculitis (Petkova et al., 2001; Roffê et al., 2016; Weaver et al., 2019).

In humans, Laguens et al. (1975) described immunoglobulins bound to the plasma membrane of both muscle fibers and endothelial cells in muscle biopsies of patients with CD. Similarly, Cossermelli et al. (1978) reported perivascular mononuclear infiltrate with deposition of IgM and C3 in the arterial wall in a patient with muscle vasculitis. Necrotizing arteritis has been reported in arteries of patients with megaesophagus (Brito and Vasconcelos, 1959), similar to that reported in mice by Roffê (Roffê et al., 2016; Weaver et al., 2019) and findings in PAN (Ozen, 2017). Our case, nonetheless, is the only one in the literature showing an AAV with autoantibodies against myeloperoxidase with documented presence of aortitis, muscle vasculitis, and probable renal involvement (Garcia-Bustos et al., 2020a). Mainly all reports share in common the predominance of mononuclear and macrophage cells, fibrinoid necrosis, and, in some cases, necrotizing vasculitis (Dias et al., 1956; Okumura et al., 1960; Cossermelli et al., 1978; Sunnemark et al., 1998; Petkova et al., 2001; Tanowitz et al., 2009; Prado et al., 2011; Roffê et al., 2016; Weaver et al., 2019). The organ involvement partially mimics that of medium and small vessel primary systemic vasculitides, including PAN or, to a lesser extent, AAV. However, there is a disagreement between human and animal studies on the importance of autoantibodies or the role of immune complexes.

As we can see, evidence on Chagas vasculitis immunopathogenesis is scarce and practically absent in humans. The role of autoreactive responses or autoantibodies is, hence, yet to be determined. Interestingly, one study found that cutaneous vasculitis in patients with systemic lupus erythematosus (SLE) was significantly associated with the presence of anti-ribosomal P protein antibodies (Shinjo and Bonfá, 2011), a commonly found autoantibody in patients with chronic CD (Mesri et al., 1990; Bonfa et al., 1993; Kaplan et al., 1997; Abraham and Derk, 2015). To date, this is the only common autoantibody in vasculitis and CD, which makes us hypothesize about its implications in the pathogeny of vasculitis associated with chronic infection by *T. cruzi*, especially after immunoglobulin deposits have been found in the vascular walls of affected patients.

## Conclusions

During the 80s and 90s, the role of autoimmunity in the pathophysiology of chronic CD was deeply studied. Whilst it is indisputable that autoimmune responses are present in patients with chronic *T. cruzi* infection mainly due to bystander activation and molecular mimicry, whether they play a key role in its pathogeny is still controversial. Vasculitis of many vascular beds is also present in these patients. However, there are practically no data on the immune processes triggering its development and we do not know if autoimmunity is present. Further studies are needed to characterize vasculitis in patients with CD and explore its underlying immune cellular and molecular pathways.

## Author Contributions

VG-B conceived the idea, searched the bibliographic materials, reviewed the existing literature, and wrote the article. MC-N aided in the search of the bibliographic materials and contributed to the writing of the article. PM, MS, and EC reviewed the literature and contributed to the writing of the article. EC supervised the work. All authors contributed to the article and approved the submitted version.

## Conflict of Interest

The authors declare that the research was conducted in the absence of any commercial or financial relationships that could be construed as a potential conflict of interest.
